# The Effects of Exendine-4 on Insulin Producing Cell Differentiation from Rat Bone Marrow-Derived Mesenchymal Stem Cells

**Published:** 2014-05-25

**Authors:** Fereshteh Nejad-Dehbashi, Mahmoud Hashemitabar, Mahmoud Orazizadeh, Somaieh Bahramzadeh, Elham Shahhosseini Pourshoushtary, Layasadat Khorsandi

**Affiliations:** 1 Cell and Molecular Research Center, Ahvaz Jundishapur University of Medical Sciences, Ahvaz, Iran; 2 Department of Anatomical Sciences, Ahvaz Jundishapur University of Medical Sciences, Ahvaz, Iran

**Keywords:** Exendin-4, Mesenchymal Stem Cells, Insulin-producing Cells, PDX-1, GLUT-2

## Abstract

**Objective:**

The aim of this study was to evaluate the effect of exendin-4 (EX-4) on differentiation of insulin-producing cells (IPCs) from rat bone marrow-derived mesenchymal stem cells (RAT-BM-MSCs).

**Materials and Methods:**

In this experimental study, RAT-BM-MSCs were cultured and the cells characterized by flow cytometry analysis of cell surface markers. RAT-BM-MSCs were subsequently treated with induction media with or without EX-4. After induction, the presence of IPCs was demonstrated with dithizone (DTZ) staining and gene expression profiles for pancreatic cell differentiation markers (PDX-1, GLUT-2, insulin) were assessed using reverse transcription polymerase chain reaction (RT-PCR). Insulin excreted from differentiated cells was analyzed with radioimmunoassay (RIA). The two-tailed student’s t-test was used for comparison of the obtained values.

**Results:**

The percentage of DTZ-positive cells significantly increased in EX-4 treated cells (p<0.05). Expression of the islet-associated genes PDX-1, GLUT-2 and insulin genes in EX-4 treated cells was markedly higher than in the cells exposed to differentiation media without EX-4. RIA analysis demonstrated significant release of insulin with the glucose challenge test in EX-4 treated cells compared to EX-4 untreated cells.

**Conclusion:**

The results of this study have demonstrated that EX-4 can enhance differentiation of IPCs from RAT-BM-MSCs.

## Introduction

Type 1 diabetes is caused by autoimmune destruction of the pancreatic islet insulin-producing β cells. Insulin administration does not prevent longterm complications of the disease as the optimal insulin dosage is difficult to adjust. Replacement of the damaged cells with regulated insulin-producing cells (IPCs) is considered the ultimate cure for type 1 diabetes. Transplantation of intact human pancreases or isolated islets has been severely limited by the scarcity of human tissue donors and the search continues for an abundant source of human IPCs. Recent progress in stem cell biology has raised hopes for the generation of regulated IPCs by differentiation from various sources of stem/progenitor cells (1, 2).

Glucagon-like peptide 1 (GLP-1) is a 30 amino acid peptide produced in intestinal L cells and released into the bloodstream in response to food intake. It is a potent incretin, in that it increases glucose-dependent secretion of insulin by pancreatic β cells. It acts directly on β cells, enhancing the effect of glucose in stimulating insulin secretion from these cells. When administered to diabetic mice, GLP-1 lowers blood glucose levels and stimulates insulin secretion (3). In addition, GLP-1 increases β cell mass by inducing the differentiation and neogenesis of ductal progenitor cells into islet endocrine cells (4, 5). It has been reported that GLP-1 is capable of enhancing fetal pig β cell differentiation from progenitor epithelial cells as well as initiating their functional maturation in isletlike cell clusters (6).

GLP-1 stimulates pro-insulin gene transcription in the pancreatic β cells, decreases gastric emptying time and reduces food intake. As a result, GLP-1 has received much attention as a possible therapeutic agent in the treatment of type II diabetes and obesity. However, GLP-1 is rapidly degraded *in vivo* by dipeptidyl peptidase IV (DPP IV) (7).

Exendin-4 (EX-4), a 39-amino acid peptide, is a GLP-1 receptor agonist that is a more potent, longer lasting insulinotropic peptide than GLP-1. The ten-fold increase in potency of EX-4 *in vivo* relative to GLP-1 is attributed to: a. increased metabolic stability as the compound is resistant to cleavage by DPP IV and many of the neutral endopeptidases that degrade GLP-1, and b. its increased affinity for the GLP-1 receptor. EX-4 is being assessed in clinical trials as a potential treatment for hyperglycemia. EX-4 and GLP-1 share a 53% amino acid sequence homology. The major difference between EX-4 and GLP-1 is in the nine amino acid C terminal sequence of EX-4, which is not present in GLP-1. Recent studies of the solution nuclear magnetic resonance (NMR) structure of the peptides show that, although both GLP-1 and EX-4 exhibit a highly helical tertiary structure, EX-4 is more stable. The helical structure of EX-4 is stabilized by the compact conformation formed by amino acids 27–39 that form a hydrophobic Trp-cage fold feature that caps and stabilizes the helix (8).

It has been previously reported that EX-4 is capable of stimulating both the differentiation of β cells from ductal progenitor cells and proliferation of β cells when administered to rats and humans (9-11).

In the present study we examined the possibility that EX-4 could enhance the differentiation of IPCs from rat bone marrow-derived mesenchymal stem cells (RAT-BM-MSCs).

## Materials and Methods

### Isolation of rat bone marrow mesenchymal stem cells

This study was approved by the Ethics Committee of Ahvaz Jundishapur University of Medical Sciences. RAT-BM-MSC cultures were prepared under sterile conditions (9). Briefly, the femur and tibia of the rats were excised with special attention given to the removal of all connective tissue attached to the bones. Bone marrow was extruded from these bones by flushing the bone marrow cavity by a syringe with an attached 20-gauge needle. The syringe was filled with culture medium (DMEM) supplemented with 10% fetal calf serum (FCS). The harvested RAT-BM-MSCs were gently pipetted to break up cell clumps in order to obtain a cell suspension. After a homogenous cell suspension was achieved, the cells were centrifuged at 1200 rpm for 7 minutes and the cell pellet was resuspended in 3 ml of culture medium. The cell suspension was seeded in 25 cm^2^ plastic tissue culture flasks with 5 ml culture medium and maintained at 37˚C in a humidified atmosphere with 5% CO2. Cultures of RAT-BM-MSCs were inspected and refed every three days and passaged when the RAT-BM-MSCs reached approximately 80% confluency. The mesenchymal population was isolated on the basis of its ability to adhere to the culture plate (12-14).

### Flow cytometry analysis

We used flow cytometry to determine expression of cell surface markers on the RAT-BM-MSCs culture prior to the use of differentiation media. Flow cytometry was performed in Department of Immunology of Ahvaz Jundishapur University of Medical Sciences. The cells were characterized with regard to a set of markers characteristic for RATBM- MSCs that included CD44, CD105, CD45, and CD34 (15).

### Induction of rat bone marrow mesenchymal stem cells to IPCs

For induction, passage-3 bone marrow-derived RAT-BM-MSCs were divided into the following groups. Group 1 was cultured in DMEM, group 2 was cultured in IPC differentiation media and we cultured group 3 in IPC differentiation media plus EX-4 (Sigma, Germany). A three-stage protocol was used to induce IPC, as follows. For stage 1, the cells (1×105/ml were cultured at 37˚C and 5% CO2 for two days in serum-free high glucose DMEM (25 mmol/L) that contained 0.5 mmol/L β-mercaptoethanol (Invitrogen, USA). In stage 2 the cells were subsequently cultured in medium that contained 1% non-essential amino acids (Invitrogen, USA), 20 ng/ml fibroblast growth factor (FGF, Sigma-Aldrich), 20 ng/ml EGF (Sigma-Aldrich), 2% B27 (Invitrogen), and 2 mmol/L L-glutamine (Hyclone Laboratories, Inc., USA) in six-well plates for eight days. For stage 3, we cultured the cells for an additional eight days in new medium that contained 10 ng/ml β-cellulin (Sigma-Aldrich), 10 ng/ ml activin A (Sigma-Aldrich), 2% B27 and 10 mmol/L nicotinamide (Sigma-Aldrich) (16). In the EX-4 group, 10 ng/ml EX-4 was added to the differentiation medium in stages 2 and 3.

### Dithizone staining

Ten mg Dithizone (Sigma-Aldrich) was completely dissolved in 10 ml of dimethyl sulfoxide (DMSO, Sigma-Aldrich,USA) and was stored at -20˚C. The working solution (pH=7.8) was prepared immediately prior to use by diluting the stock solution (1:10) in PBS. For each dish, 2 ml of the DTZ solution were added and allowed to incubate for 30 minutes at 37˚C. Average percentages of cells that stained with DTZ were calculated by dividing the number of DTZ positive cells in a random microscopic field by the total number of cells in the same field, after which the result was multiplied by 100. For each culture the mean of three fields was considered (17, 18).

### RNA preparation and reverse transcription polymerase chain reaction

Using the RNeasy Mini Kit Qiagen, Valencia, CA, USA), RNA was isolated from the harvest cells according to the manufacturer’s instructions. Reverse transcription polymerase chain reaction (RT-PCR) was performed using a One- Step RT-PCR Kit (Qiagen, Valencia, CA, USA) which contains reverse transcriptase to synthesize cDNA from the isolated RNA and DNA polymerase for the PCR. RT-PCR conditions consisted of a 30 minute step at 50˚C to allow for reverse transcriptase activity followed by 15 minutes at 95˚C to deactivate the reverse transcriptase and activate Taq polymerase present in the enzyme mixture. The PCR process consisted of 6 seconds at 94˚C (denaturing step), 30 seconds at the annealing temperature (55˚C), and a 45 second step at 72˚C for extension with all steps repeated for 30 cycles. A final extension step lasted 10 minutes at 72˚C.

Primer sequences were as follows with the expected product length: PDX-1, sense 5´ AAACGCCACACACAAGGAGAA 3´ and antisense 5´ AGACCTGGCGGTTCACATG 3´ (150 bp); GLUT-2, sense 5´ CAGCTGTCTCTGTGCTGCTTGT 3´ and antisense 5´ GCCGTCATGCTCACATAACTCA 3´ (150 bp); insulin, sense 5´ TCTTCTACACACCCATGTCCC 3´ and antisense 5´ GGTGCAGCACTGATCCAC 3´, (149 bp). GAPDH, sense 5´CTC TGGTGGACCTCATGGCCTAC 3´ and antisense 5´ CAGCAACTGAGGGCCTCTCT 3´ (105 bp), was used as the housekeeping gene (19).

### Radioimmunoassay

The differentiated cells were pre-incubated for one hour in glucose-free Krebs-ringer bicarbonate (KRB) and incubated with KRB that contained 5.56 mmol/L, 16.7 mmol/L or 25 mmol/L of glucose (glucose challenge) for an additional one hour, respectively. The KRB media were collected and frozen at -80˚C until assayed (20). The insulin assay was performed by radioimmunoassay (RIA) using a commercially available rat RIA kit (Millipore) according to the manufacturer’s instructions. Determinations were carried out in triplicate and the means and standard deviations were obtained.

### Statistical analysis

A two-tailed student’s t test was used for comparing the obtained values. For statistical purposes at least three independent cultures were considered. All values have been stated as means ± standard deviations. P<0.05 was considered to be statistically significant.

## Results

**Fig 1:**
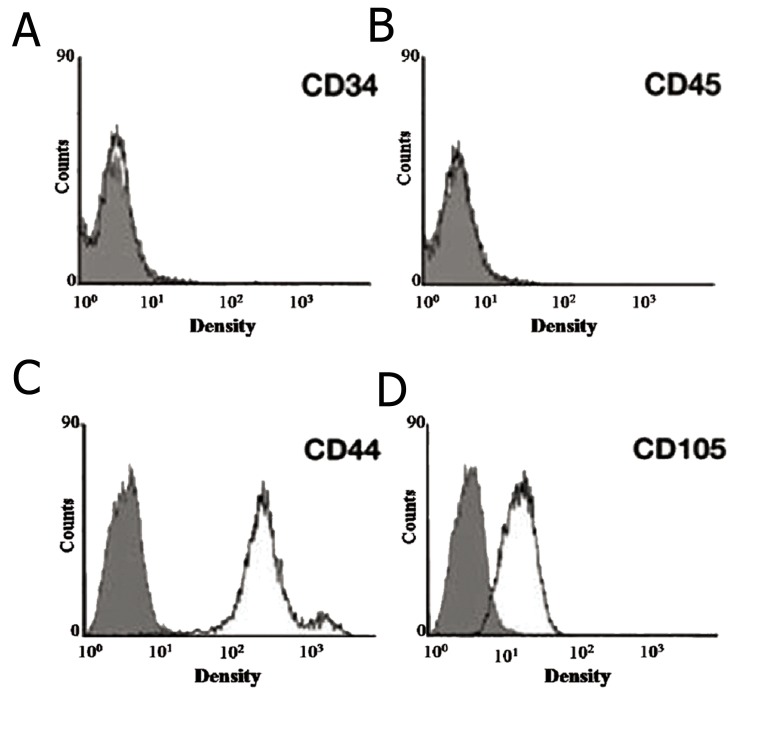
Characterization of different surface markers: CD34, CD45, CD44 and CD1O5. High expression of CD44 and CD105, low expression of CD34 and no expression of CD45 are shown. Gray and white histograms show control and cell surface markers.

Cell surface markers detected by flow cytometry revealed that RAT-BM-MSCs highly expressed CD105 and CD44, whereas there were no expressions of CD34 and CD45 detected (Fig 1).

### Morphological changes of rat bone marrow mesenchymal stem cell differentiation

**Fig 2:**
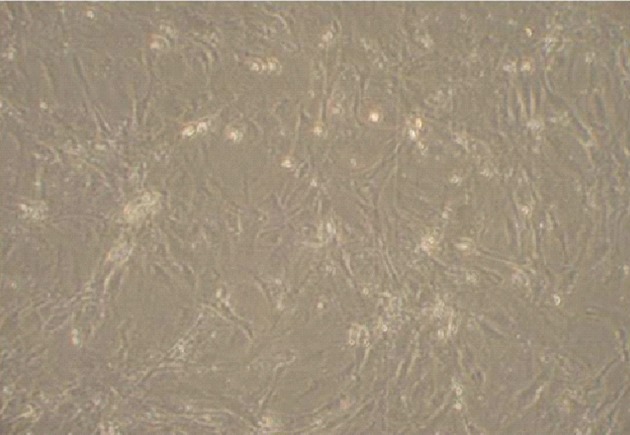
Rat bone marrow mesenchymal stem cells (RAT-BMMSCs) at passage 3. Spindle shape cells are observed. Magnification: ×400.

**Fig 3:**
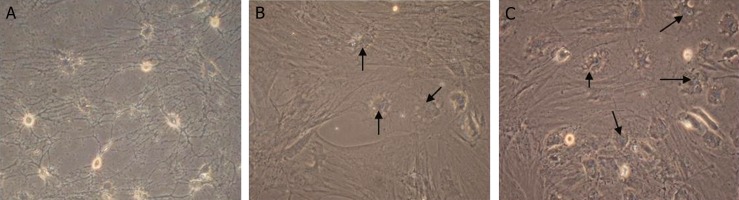
Isolation and characterization of rat bone marrow mesenchymal stem cells (RAT-BM-MSCs). A. Morphological changes of undifferentiated RAT-BM-MSCs in DMEM. B. Morphological changes of RAT-BM-MSCs differentiation to IPCs in IPC differentiation media without EX-4. C. Morphological changes of RAT-BM-MSCs in IPC differentiation media with EX-4. Magnification: ×400.

Under an inverted microscope, undifferentiated RAT-BM-MSCs were typical of adherent spindle and fibrocyte-like cells at passage 3 (Fig 2). The RAT-BM-MSCs cultured in undifferentiaton media (control group) showed various shapes including spherical, neuron-like cells or glial-like cells (Fig 3A). Under differentiation media with EX-4, the RAT-BMMSCs forming spherical type with confluence similar to pancreatic islet-like cells. Round shape morphology in differentiation media without EX-4 were lesser than those exposed to differentiation media containing EX-4 (Fig 3B, C).

### Dithizone staining

**Fig 4:**
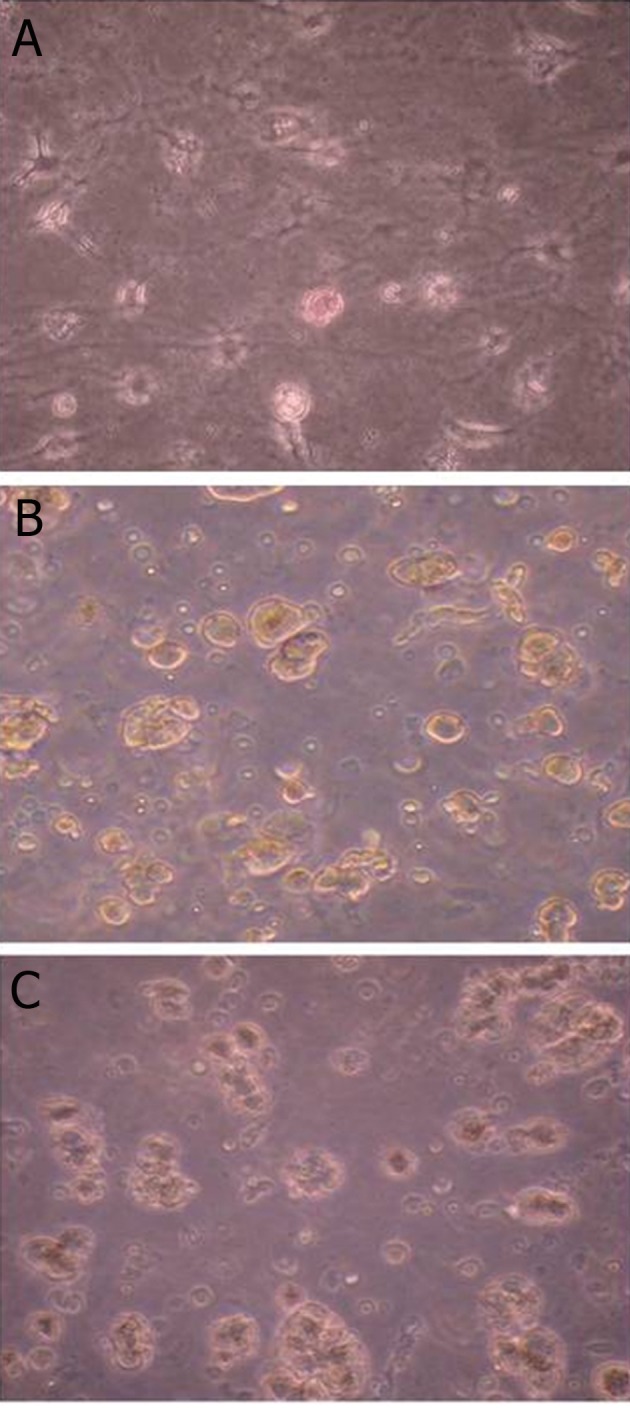
DTZ staining of RAT-BM-MSCs. A. Spontaneously differentiated rat bone marrow mesenchymal stem cells (RAT-BM-MSCs) in DMEM stained positive for dithizone (DTZ). B. DTZ-positive cells in insulin-producing cell (IPC) differentiation media without exendin-4 (EX-4). C. DTZpositive RAT-BM-MSCs) in IPC differentiation media with EX-4. Magnification: ×400.

**Fig 5:**
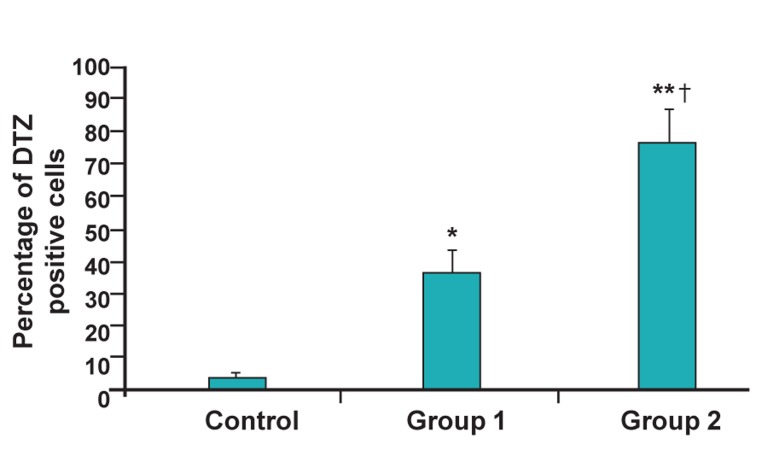
Percentage of dithizone (DTZ) staining in various groups. Values are expressed as mean ± SD. *; p<0.01, **; p<0.001, ; p<0.001, * and ; Compared to control and group 1, respectively

To verify the insulin expression in the differentiated cells, dithizone which specifically stains insulin granules present in β-cell was used. As shown in figure 4, most of the cells in IPC differentiation media were positive for dithizone staining, especially in presence of EX-4, The percentage of dithizone positive cells were significantly increased in group IPC differentiation media without EX-4 (group 1) and with EX-4 (group 2) compared to control group. The percentage of stained cells (Fig 5) in group 2 were significantly higher than group 1 (p<0.05).

### Gene expression of bone marrow-derived IPCs

**Fig 6:**
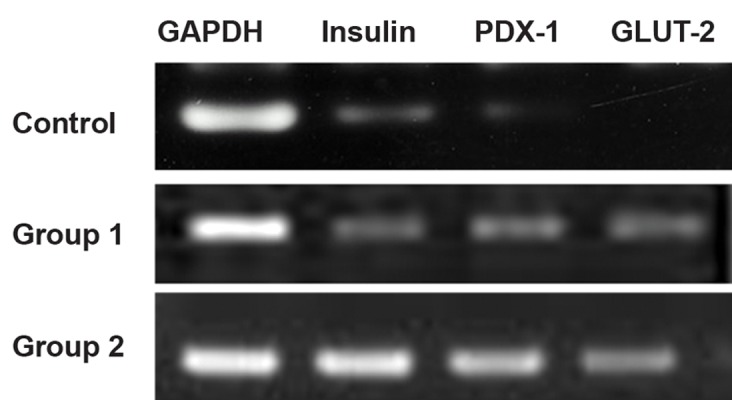
Expression of genes encoded in β cell markers of various groups.

To determine whether RAT-BM-MSCs had undergone pancreatic differentiation, we assessed gene expression profiles for pancreatic cell differentiation markers by RT-PCR. As illustrated in figure 6, low expression levels of PDX-1, GLUT-2 and insulin was detected in undifferentiated RAT-BM-MSCs (control). In RAT-BM-MSCs treated by differentiation media without EX-4 (group 1), expression of these genes was markedly higher in compare to control. In EX-4 treated cells expression of these genes were higher than group 1.

### Insulin release in response to glucose stimulation

**Fig 7:**
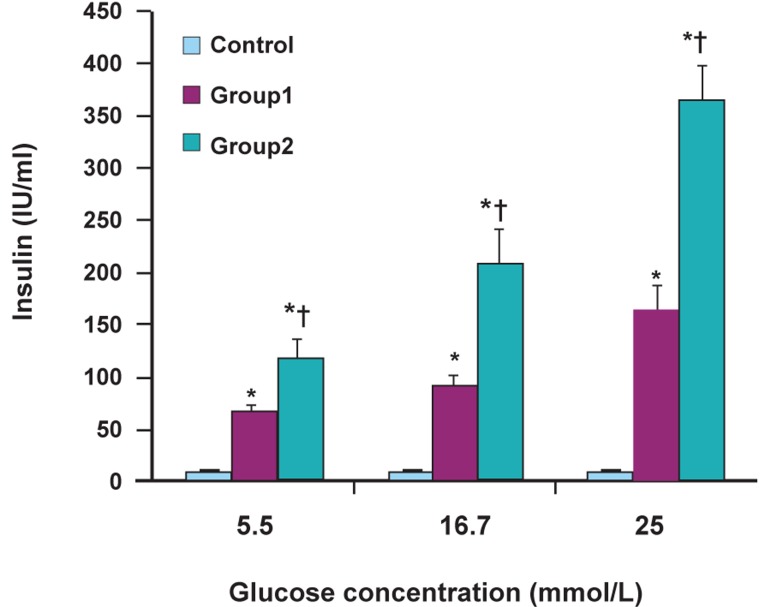
Insulin excretion changes in various groups. Values are expressed as mean ± SD. *; p<0.001, ; p<0.001, * and : Compared to control and group 1, respectively.

Cultured RAT-BM-MSCs in the control group showed no significant release of insulin in the presence or absence of the glucose challenge. The differentiated cells in the absence of EX-4 released insulin at a low concentration of glucose (5.56 mmol/L) and released approximately 2.5 fold insulin under glucose challenge (25 mmol/L;, p<0.01). There was significantly more insulin secretion of differentiated cells in the presence of EX-4 at a low concentration of glucose and under glucose challenge compared to untreated EX-4 cells (p<0.01). The results of RIA are depicted in figure 7.

## Discussion

Transplantation of pancreatic islet cells and utilization of stem cells as a potential cure for diabetes mellitus have become the subjects of intense interest and activity over the past several years (21-23). However, some obstacles, such as limited supply of human islet tissue, immune rejection, and ethical issues remain. Bone marrow has been known for years as a safe, abundant source for large quantities of adult stem cells (24). In the present study we have demonstrated that EX-4 affected the transdifferentiation process of RAT-BM-MSCs cells to IPCs. Park et al. (25) showed that EX-4 and exercise promoted β cell function and mass in islets of diabetic rats. Stoffers et al. (26) reported that exposure to EX-4 in the newborn period reversed the adverse consequences of fetal programming and prevented the development of diabetes in adulthood. It has been revealed that GLP-1 promotes the expansion of pancreatic β cell mass by stimulating neogenesis as well as proliferation of existing β cells (27-29). Administration of the long-acting GLP-1 analog EX-4 during regeneration after 90% partial pancreatectomy in rats has resulted in a sustained improvement in glucose homeostasis associated with a 40% increase in β cell mass due to increases in both neogenesis and replication (3). Further, chronic treatment of adult diabetic mice with either GLP-1 or EX-4 also improves glucose tolerance, increases islet size, and stimulates pancreatic duodenal homeobox (PDX) protein expression in the pancreas (10).

In this study the existence of IPCs was confirmed by DTZ staining and expression pattern analysis of islet-specific genes. We have shown that expression of PDX-1 in EX-4 treated cells markedly increased. PDX is a pancreatic homeoprotein critical for early development of both the endocrine and exocrine pancreas. It mediates glucose-responsive stimulation of insulin gene transcription (30). PDX-1 plays a crucial role in the control of several genes expressed in the pancreas. Its capacity to activate gene transcription in a tissue specific mode is dependent on its ability to interact with other transcription factors (31, 32).

PDX-1 binds and transactivates the promoters of several physiologically relevant genes within the β cell, including insulin, glucose transporter 2 (GLUT-2), glucokinase, and islet amyloid polypeptide (33). There were elevated expressions of insulin 2 and GLUT-2 genes in EX-4 treated cells in the present study. It has been reported that expressions of these genes indicate differentiation and fully functional IPCs. Additionally, RIA analysis has demonstrated significant expression of insulin upon glucose challenge in EX-4 treated cells compared to untreated cells.

In pancreatic β cells, glucose uptake is controlled by GLUT-2, which is essential in the mechanism of glucose-induced insulin secretion (34). GLUT-2 is the glucose sensor of β cells that leads to the production of insulin (35). GLP-1 increases insulin secretion and the biosynthesis of important β cell products in addition to insulin such as glucokinase and GLUT-2 glucose transporters (36). The present study has also detected insulin gene expression in non-induced cells (control), which indicated that RAT-BM-MSCs could spontaneously differentiate into IPCs. This result supported previous findings that adult stem cells could spontaneously differentiate (37).

## Conclusion

This study has demonstrated that EX-4 can enhance the differentiation of RAT-BM-MSCs into insulin producing cells. However, further studies are needed to understand the mechanism of action of EX-4 on MSCs.
